# Comparative study of breast cancer with or without concomitant Paget disease: An analysis of the SEER database

**DOI:** 10.1002/cam4.2242

**Published:** 2019-05-28

**Authors:** Shijing Chen, Huaquan Chen, Ying Yi, Xuemei Jiang, Hai Lei, Xue Luo, Yu Chen, Sha Liu, Dan Yuan, Xinjian Jia, Junyan Li

**Affiliations:** ^1^ Department of Dermatology People's Hospital of DeYang City Deyang China; ^2^ Department of Br Surgery People's Hospital of DeYang City Deyang China

**Keywords:** Breast Cancer, Ductal Carcinoma In Situ, Infiltrating Ductal Carcinoma, Mammary Paget Disease, Surveillance, Epidemiology, and End Results

## Abstract

**Background:**

Most mammary Paget disease (MPD) is associated with underlying in situ or invasive breast cancer. The objective of this study was to compare the clinicopathological characteristics and survival outcomes between breast cancer with Paget disease (PD) and breast cancer alone.

**Methods:**

From the Surveillance, Epidemiology, and End Results (SEER) database, 2000‐2015, of the US National Cancer Institute, we identified 1569 women who had PD with invasive ductal carcinoma (PD‐IDC) and 1489 women who had PD with ductal carcinoma in situ (PD‐DCIS). Independent demographic and clinicopathological variables as well as survival outcomes of these patients were compared to patients with the corresponding breast cancer without concomitant PD.

**Results:**

PD‐IDC and PD‐DCIS both had worse survival outcomes and poorer tumor characteristics than the corresponding disease without PD. Contrary to in the breast cancer alone groups, in the breast cancer with PD groups, the HR status (*P* = 0.182 in PD‐IDC and *P* = 0.371 in PD‐DCIS), HER2 status (*P* = 0.788 in PD‐IDC and *P* = 0.643 in PD‐DCIS), and combined molecular subtype (*P* = 0.196 in PD‐IDC and *P* = 0.853 in PD‐DCIS) were not found to affect disease prognosis. After matching tumor characteristics and treatment approaches, PD‐IDC as well as PD‐DCIS exhibited no significant difference in disease prognosis with corresponding IDC and DCIS. Finally, by comparative analysis, a kind of PD‐DCIS (ICD‐O‐3 code 8543/3) showed many invasive behaviors (31.8% of 8543/3 patients had stage I‐III cancer) and was associated with worse survival outcomes than the other type of PD‐DCIS.

**Conclusions:**

Breast cancer with concomitant PD was associated with more aggressive tumor characteristics and worse survival outcomes. The HR status, HER2 status, and combined molecular subtype could not affect the prognosis of breast cancer with PD. Moreover, a portion of the PD‐DCIS cases were invasive breast cancer cases that required special treatment.

## 1 INTRODUCTION

Paget disease (PD) is a rare cutaneous adenocarcinoma that targets mainly the nipple‐areola complex (NAC) of the breast as well as the genital and perianal skin.^1^ It is named after Sir James Paget, who reported that all of his patients developed breast cancer within 2 years after nipple changes was observed. Later, studies showed that approximately 82%‐100% of mammary Paget disease (MPD) cases are associated with underlying in situ or invasive breast cancer,^2^ and this observation supports the most accepted pathogenesis theory, which posits that Paget cells are ductal carcinoma cells that have migrated from the underlying ducts to the epidermis.^3^, ^4^

According to underlying malignancy, MPD can be divided into three groups: PD with invasive ductal carcinoma (PD‐IDC), PD with ductal carcinoma in situ (PD‐DCIS), and PD of the nipple without concurrent breast cancer.^4^ A previous study suggested that compared with IDC without PD, PD‐IDC has more aggressive pathology characteristics, including higher grade, more advanced tumor stage, hormone receptor (HR)‐negative status and human epidermal growth factor receptor 2 (HER2/neu)‐positive status,^4^, ^5^, ^6^ which may lead to poor prognostic outcomes and reduced survival.^7^, ^8^, ^9^ Based on the National Cancer Institute's Surveillance, Epidemiology, and End Results (SEER) data, PD‐IDC has been found to be associated with worse prognosis than IDC alone,^10^ and among the three subtypes of PD, PD‐IDC has the worst prognosis.^7^ Nevertheless, little is known about PD‐DCIS clinicopathologic features and survival outcomes, especially the differences in these characteristics compared with those of DCIS alone.^11^ Furthermore, how do these differences arise? Do these differences indicate a distinctive property and origin of MPD? There is a lack of relevant research into both questions.

In this study, we used population‐based data to examine the effect of MPD on the clinicopathological characteristics and survival outcomes of both IDC and DCIS, further exploring the properties and origin of MPD.

## 2 MATERIALS AND METHODS

### 2.1 Data source

The SEER database contains cancer incidence and mortality data from 18 population‐based registries that represent approximately 30% of the US population. We obtained data from the SEER database based on the November 2017 submission by using SEER*Stat software version 8.3.5.

### 2.2 Patient selection

We used International Classification of Diseases for Oncology, third edition histology and behavior codes (ICD‐O‐3 Hist/behav) to identify patients. Based on the underlying breast cancer subtype, we examined two retrospective cohorts. Cohort 1 consisted of a PD‐IDC group (ICD‐O‐3 code 8541/3: Paget disease and infiltrating ductal carcinoma of the breast) and an IDC group (ICD‐O‐3 code 8500/3: Infiltrating ductal carcinoma). Cohort 2 comprised a PD‐DCIS group (ICD‐O‐3 code 8543/2: Paget disease in situ and intraductal carcinoma and 8543/3: Paget disease and intraductal carcinoma) and a DCIS group (ICD‐O‐3 code 8050/2: Papillary carcinoma in situ; 8201/2: Cribriform carcinoma in situ; 8230/2: Ductal carcinoma in situ, solid type; 8500/2: Intraductal carcinoma, noninfiltrating; 8501/2: Comedocarcinoma, noninfiltrating; 8503/2: Noninfiltrating intraductal papillary adenocarcinoma; 8507/2: Intraductal micropapillary carcinoma; and 8523/2: Intraductal with other types of carcinoma in situ). Other selection criteria included female sex, diagnosed between 2000 and 2015, no prior history of any cancer, no distant metastases. The exclusion criteria were as follows: (a) age at diagnosis younger than 18 years; (b) TNM stage unknown; (c) incomplete survival data and follow‐up information; and (d) no surgery performed.

### 2.3 Study variables

Our main outcome of interest was survival. Overall survival (OS) and breast cancer‐specific survival (BCSS: The percentage of patients who have not died from breast cancer, by NCI Dictionary of Cancer Terms) were calculated from the date of diagnosis to the last date of available vital status data. We also evaluated independent demographic and clinicopathological variables for each case, including age, race (white, black, and Hispanic/other/unknown), tumor location (classified as central/NAC or by quadrant), histological grade (grade 1, 2, 3, and 4), TNM stage (Adjusted AJCC sixth edition T, N, and M stages), HR status (estrogen receptor (ER)+/progesterone receptor (PR)+, ER+/PR‐, ER‐/PR+, and ER‐/PR‐), HER2/neu status, molecular subtype (Her2‐/HR+, Her2+/HR+, Her2+/HR‐, and Triple Negative), type of surgery (breast‐conserving surgery and mastectomy), radiotherapy use, and chemotherapy use.

### 2.4 Statistical analysis

For the demographic and clinicopathological data, continuous variables such as age were compared using Student's *t* test, and categorical variables were compared using Pearson's chi‐square test. Survival curves were generated according to the Kaplan‐Meier method and compared using the log‐rank test. Univariate and multivariate Cox proportional hazards regression models were constructed to analyze factors associated with survival in the patients with or without PD. Then, we performed Propensity Score Matching (PSM) to further evaluate the effect of PD on survival by adjusting for age, tumor location, tumor grade, T stage, N stage, HR status, HER2/neu status, and type of therapy (surgery, radiation, and chemotherapy). Statistical significance was set as a two‐sided *P*‐value < 0.05, and all confidence intervals (CIs) are reported at the 95% confidence level. All the statistical analyses were performed using SPSS version 25.0 statistical software (IBM Corp, Armonk, NY).

## 3 RESULTS

In total, cohort 1 consisted of 1569 women who had PD‐IDC and 467 004 control group women who had IDC alone, while cohort 2 contained 1489 women who had PD‐DCIS and 144 699 control group women who had DCIS alone (Figure S1).

### 3.1 Clinicopathological characteristics

The demographics and clinicopathological characteristics of the two cohorts are summarized in Table 1.

**Table 1:** Patient characteristics

Clinical characteristics	No. of Patients (%)		P*	No. of Patients (%)		P**
	PD‐IDC, n = 1569	IDC, n = 467 004		PD‐DCIS, n = 1489	DCIS, n = 144 699	
Age at diagnosis Mean ± SD, y	59.91 ± 15.286	59.36 ± 13.531	0.158	63.17 ± 14.611	58.84 ± 12.154	<0.001
Race			<0.001			<0.001
White	1016 (64.8%)	330 024 (70.7%)		1234 (82.9%)	112 381 (77.7%)	
Black	190 (12.1%)	47 599 (10.2%)		128 (8.6%)	15 720 (10.9%)	
Hispanic/Other/Unknown	363 (23.1%)	89 381 (19.1%)		127 (8.5%)	16 598 (11.5%)	
Laterality			0.441			0.011
Left	809 (51.6%)	236 354 (50.6%)		811 (54.5%)	73 983 (51.1%)	
Right	759 (48.4%)	230 572 (49.4%)		678 (45.5%)	70 673 (48.9%)	
Tumor location			<0.001			<0.001
Central/NAC	440 (38.2%)	24 465 (5.9%)		949 (78.4%)	10 564 (8.7%)	
Upper inner quadrant	76 (6.6%)	56 602 (13.7%)		15 (1.2%)	12 411 (10.3%)	
Lower inner quadrant	61 (5.3%)	27 422 (6.6%)		26 (2.1%)	9302 (7.7%)	
Upper outer quadrant	243 (21.1%)	169 194 (40.9%)		66 (5.5%)	47 270 (39.1%)	
Lower outer quadrant	70 (6.1%)	34 087 (8.2%)		31 (2.6%)	10 207 (8.4%)	
Overlapping lesion	262 (22.7%)	101 917 (24.6%)		123 (10.2%)	31 167 (25.8%)	
Tumor grade			<0.001			<0.001
Grade I	101 (6.4%)	92 245 (19.8%)		21 (1.9%)	17 236 (13.9%)	
Grade II	502 (32.0%)	192 403 (41.2%)		168 (15.6%)	51 112 (41.1%)	
Grade III	934 (59.5%)	178 023 (38.1%)		640 (59.4%)	42 609 (34.2%)	
Grade IV	32 (2.0%)	4333 (0.9%)		248 (23.0%)	13 476 (10.8%)	
AJCC sixth edition stage			<0.001			<0.001
0	0 (0%)	0 (0%)		1149 (83.1%)	144 699 (100.0%)	
I	555 (35.6%)	236 401 (50.6%)		133 (9.6%)	0 (0%)	
II	547 (35.1%)	174 292 (37.3%)		72 (5.2%)	0 (0%)	
III	457 (29.3%)	56 299 (12.1%)		28 (2.0%)	0 (0%)	
T			<0.001			<0.001
T0	0 (0%)	0 (0%)		1151 (83.3%)	144 699 (100.0%)	
T1	801 (51.5%)	297 117 (63.7%)		149 (10.8%)	0 (0%)	
T2	477 (30.7%)	139 192 (29.9%)		44 (3.2%)	0 (0%)	
T3	113 (7.3%)	19 414 (4.2%)		19 (1.4%)	0 (0%)	
T4	163 (10.5%)	10 578 (2.3%)		19 (1.4%)	0 (0%)	
N			<0.001			<0.001
N0	785 (50.0%)	313 992 (67.2%)		1340 (97.4%)	144 699 (100.0%)	
N1	471 (30.0%)	109 931 (23.5%)		26 (1.9%)	0 (0%)	
N2	194 (12.4%)	28 708 (6.1%)		7 (0.5%)	0 (0%)	
N3	119 (7.6%)	14 373 (3.1%)		3 (0.2%)	0 (0%)	
Hormone receptor status			<0.001			<0.001
ER+/PR+	498 (35.5%)	291 341 (66.6%)		181 (19.7%)	70 545 (74.4%)	
ER+/PR‐	235 (16.7%)	50 225 (11.5%)		143 (15.6%)	10 159 (10.7%)	
ER‐/PR+	49 (3.5%)	5741 (1.3%)		31 (3.4%)	817 (0.9%)	
ER‐/PR‐	622 (44.3%)	90 020 (20.6%)		564 (61.4%)	13 351 (14.1%)	
HER2 status^a^			<0.001			<0.001
Positive	254 (63.5%)	30 223 (16.7%)		84 (83.2%)	2313 (32.4%)	
Negative	146 (36.5%)	151 053 (83.3%)		17 (16.8%)	4831 (67.6%)	
Molecular subtype^a^			<0.001			<0.001
HR+/Her2‐	121 (30.5%)	128 129 (70.8%)		10 (10.0%)	4405 (62.0%)	
HR+/Her2+	132 (33.2%)	21 112 (11.7%)		25 (25.0%)	1544 (21.7%)	
HR‐/Her2+	119 (30.0%)	9064 (5.0%)		58 (58.0%)	752 (10.6%)	
HR‐/Her2‐	25 (6.3%)	22 767 (12.6%)		7 (7.0%)	400 (5.6%)	
Surgery			<0.001			<0.001
Partial mastectomy	171 (10.9%)	277 695 (59.6%)		483 (32.6%)	103 613 (71.8%)	
Mastectomy	1394 (89.1%)	188 167 (40.4%)		1000 (67.4%)	40 720 (28.2%)	
Chemotherapy			<0.001			<0.001
Yes	841 (53.6%)	206 903 (44.3%)		53 (3.6%)	1038 (0.7%)	
No	728 (46.4%)	260 101 (55.7%)		1436 (96.4%)	143 661 (99.3%)	
Radiotherapy			<0.001			<0.001
Yes	425 (27.1%)	252 220 (54.0%)		297 (19.9%)	67 811 (46.9%)	
No	1144 (72.9%)	214 784 (46.0%)		1192 (80.1%)	76 888 (53.1%)	

Abbreviations: AJCC, American Joint Committee on Cancer; DCIS, ductal carcinoma in situ; ER, estrogen receptor; HER2, human epidermal receptor‐2; HR, Hormone receptor; IDC, invasive ductal carcinoma; NAC, nipple‐areola complex; PD‐DCIS, Paget disease with ductal carcinoma in situ; PD‐IDC, Paget disease with invasive ductal carcinoma; PR, progesterone receptor; +, positive; and ‐, negative

a HER2 status and molecular subtype data were available for only the patients who were diagnosed between 2010 and 2015.

In cohort 1, the PD‐IDC patients and the IDC alone patients were similar in age at diagnosis and laterality. However, the patients who had PD‐IDC were more likely to have more aggressive pathology characteristics, such as a higher histologic grade (61.5% of PD‐IDC patients vs 39.0%, of IDC alone patients had Grade III/IV lesions, *P* < 0.001), more advanced AJCC stage (29.3% vs 12.1%, respectively, had stage III disease, *P* < 0.001), lower HR‐positive ratio (55.7% vs 79.4%, respectively, had ER + or PR + cancer, *P* < 0.001), and higher HER2‐positive ratio (63.5% vs 16.7%, respectively, *P* < 0.001), than the IDC alone patients. With respect to treatment, compared with the IDC alone group patients, the PD‐IDC group patients were more likely to have received a total mastectomy without radiotherapy over a partial mastectomy with radiotherapy (*P* < 0.001).

In cohort 2, for PD‐DCIS and DCIS, all measured variables were significantly different, and PD‐DCIS tumors showed a more aggressive manner than DCIS tumors as well. Furthermore, a small portion of PD‐DCIS tumors even demonstrated some invasiveness (16.9% of PD‐DCIS tumors vs 0% of DCIS tumors were stage I‐III carcinoma, *P* < 0.001).

### 3.2 Survival analyses

#### 3.2.1 Kaplan‐Meier analyses for survival

**Figure 1:**
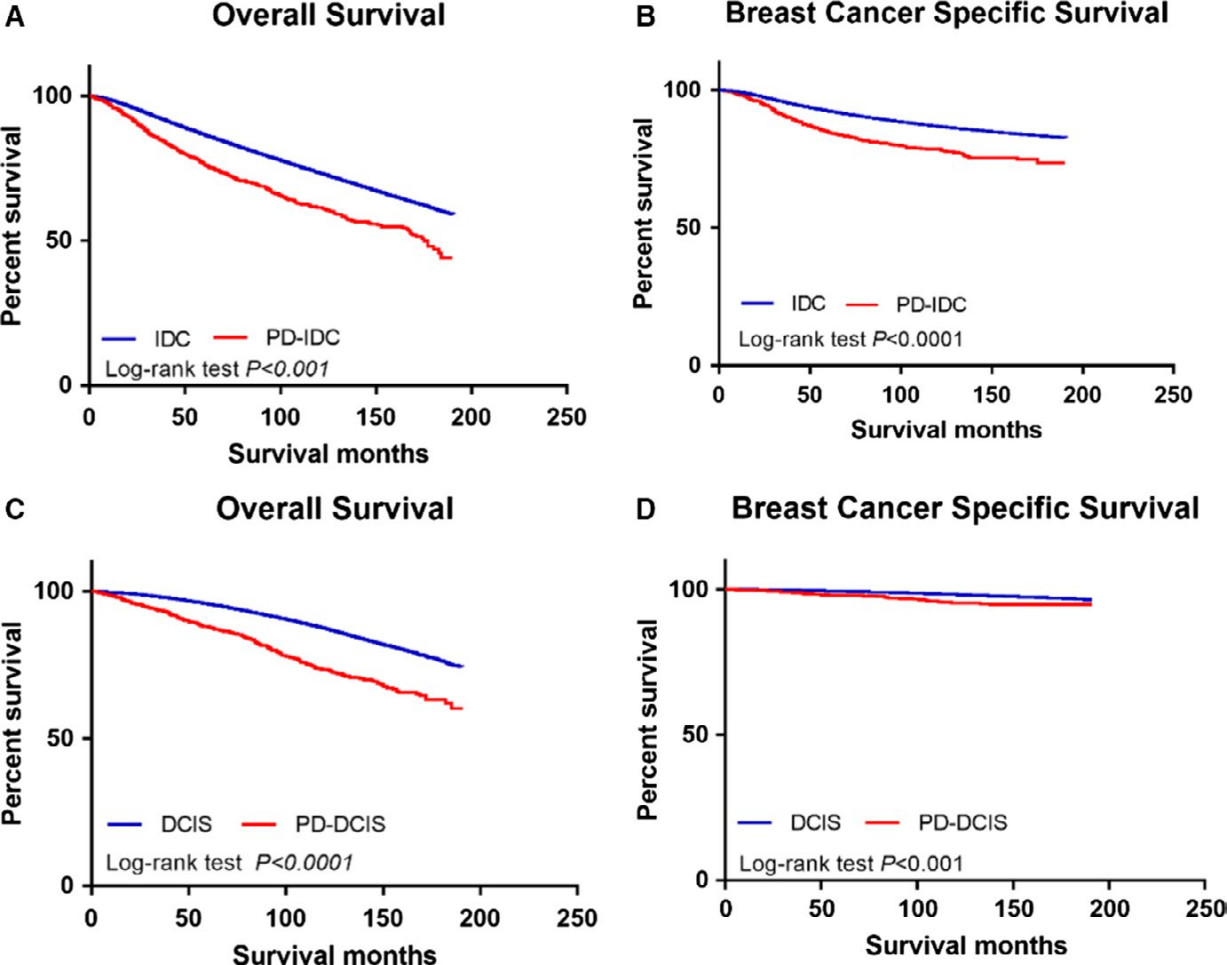
Kaplan‐Meier curves without adjusting for tumor characteristics and treatment approaches. (A) Overall survival of the PD‐IDC group and the IDC group. (B) Disease‐specific survival of the PD‐IDC group and IDC group. (C) Overall survival of the PD‐DCIS group and the DCIS group. (D) Disease‐specific survival of the PD‐DCIS group and the DCIS group

In cohort 1, the median length of follow‐up was 71 months for the PD‐IDC group and 68 months for the IDC group (*P* = 0.316). Kaplan‐Meier curves comparing the survival times of the two groups are presented in Figure 1A and 1. The patients with PD‐IDC had significantly worse survival, with a 5‐year OS rate of 76.2% for the PD‐IDC group vs 86.6% for the IDC group and a 10‐year survival rate of 61.5% for the PD‐IDC group vs 73.4% for the IDC group (*P* < 0.001). For BCSS, the 5‐year and 10‐year survival rates were 84.9% and 78.5%, respectively, in the PD‐IDC group and 92.4% and 86.9%, respectively, in the IDC alone group (*P* < 0.001).

In cohort 2, the median follow‐up time was similar in the two groups (PD‐DCIS: 81 months, DCIS: 79 months, *P* = 0.446), and the PD‐DCIS patients showed a lower survival rate than the DCIS patients. The 5‐year and 10‐year OS rates for the PD‐DCIS group were 88.6% and 74.4%, respectively, and the corresponding survival rates for the DCIS group were 95.6% and 87.3% (*P* < 0.001)(Figure 1C). In addition, the 5‐year BCSS rates were 98.2% and 99.4% in the PD‐DCIS group and the DCIS group, respectively, while the 10‐year BCSS rates were 96.3% and 98.3% in the PD‐DCIS group and the DCIS group, respectively (*P* < 0.001) (Figure 1D). The survival comparison of the two PD‐DCIS types is presented in Figure S1 and shows that the prognosis of the 8543/3 group was worse than that of the 8543/2 group.

#### 3.2.2 Cox proportional hazards models for mortality

Table 2 shows the log‐rank test results of different indicators associated with the breast cancer‐specific mortality of breast cancer. For PD‐IDC, the significant factors were age at diagnosis (*P* < 0.001), race (*P* = 0.021), tumor location (*P* = 0.012), tumor grade (*P* < 0.001), AJCC stage (*P* < 0.001), T stage (*P* < 0.001), N stage (*P* < 0.001), surgery type (*P* = 0.004), and radiotherapy use (*P* = 0.049). In contrast to the results for the IDC group, HR status, HER2 status, and molecular subtype were not associated with prognosis in the PD‐IDC group. For PD‐DCIS, only age at diagnosis (*P* < 0.001), tumor grade (*P* = 0.005), AJCC stage (*P* < 0.001), T stage (*P* = 0.002), N stage (*P* < 0.001), and chemotherapy use (*P* = 0.013) significantly affected mortality, and these prognostic factors differed from those for the DCIS group.

**Table 2:** Survival analyses—univariate analyses

Variable	Category	*P*			
		PD‐IDC	IDC	PD‐DCIS	DCIS
Age at diagnosis (years)		<0.001	<0.001	<0.001	<0.001
Race	White	0.021	<0.001	0.134	<0.001
	Black				
	Hispanic/Other/Unknown				
Laterality	Left	0.619	0.004	0.070	0.442
	Right				
Tumor location	Central/NAC	0.012	<0.001	0.192	0.009
	Upper inner quadrant				
	Lower inner quadrant				
	Upper outer quadrant				
	Lower outer quadrant				
	Overlapping lesion				
Tumor grade	Grade I	<0.001	<0.001	0.005	0.006
	Grade II				
	Grade III				
	Grade IV				
AJCC sixth edition stage	0	<0.001	<0.001	<0.001	—
	I				
	II				
	III				
T	T0	<0.001	<0.001	0.002	—
	T1				
	T2				
	T3				
	T4				
N	N0	<0.001	<0.001	<0.001	—
	N1				
	N2				
	N3				
Hormone receptor status	ER+/PR+	0.182	<0.001	0.371	<0.001
	ER+/PR‐				
	ER‐/PR+				
	ER‐/PR‐				
HER2 status^a^	Positive	0.788	0.013	0.643	0.692
	Negative				
Molecular subtype^a^	HR+/Her2‐	0.196	<0.001	0.853	0.069
	HR+/Her2+				
	HR‐/Her2+				
	HR‐/Her2‐				
Surgery	Partial mastectomy	0.004	<0.001	0.530	0.060
	Mastectomy				
Chemotherapy	Yes	0.404	<0.001	0.013	<0.001
	No				
Radiotherapy	Yes	0.049	<0.001	0.369	<0.001
	No				

Abbreviations: AJCC, American Joint Committee on Cancer; DCIS, ductal carcinoma in situ; ER, estrogen receptor; HR, Hormone receptor; HER2, human epidermal receptor‐2; IDC, invasive ductal carcinoma; NAC, nipple‐areola complex; PD‐DCIS, Paget disease with ductal carcinoma in situ; PD‐IDC, Paget disease with invasive ductal carcinoma; PR, progesterone receptor; +, positive; and −, negative

a HER2 status and molecular subtype data were available for only the patients who were diagnosed between 2010 and 2015.

Next, we performed multivariate Cox proportional hazards regression analysis with the variables with *P*‐values less than 0.1 in the univariate analyses to compute hazard ratios and 95% CIs, (Table 3). In the PD‐IDC group, the significant indicators of disease‐specific mortality were age at diagnosis (*P* < 0.001), race (*P* = 0.018), tumor location (*P* = 0.024), tumor grade (*P* = 0.001), T stage (*P* < 0.001), and N stage (*P* < 0.001). In the PD‐DCIS group, the variables that had prognostic significance were age at diagnosis (*P* < 0.001), T stage (*P* = 0.005), and N stage (*P* < 0.001). In addition, among the 1305 PD‐DCIS patients with sufficient information, only 36 patients died of breast cancer, which may influence the reliability of the multivariate analysis.

**Table 3:** Survival analyses—multivariate analyses of Paget disease

	Variable	Category/Mean	Hazard ratio	95% Confidence interval	*P*‐value
PD‐IDC	Age at diagnosis (years)	Mean: 60.07 y	1.034	1.022‐1.045	<0.001
	Race	White	1	Referent	0.018
		Black	1.285	0.842‐1.961	
		Hispanic/Other/Unknown	0.618	0.410‐0.931	
	Tumor location	Central/NAC	1	Referent	0.024
		Upper inner quadrant	1.619	0.914‐2.867	
		Lower inner quadrant	2.641	1.498‐4.656	
		Upper outer quadrant	1.399	0.919‐2.130	
		Lower outer quadrant	1.712	0.947‐3.097	
		Overlapping lesion	1.252	0.821‐1.909	
	Tumor grade	Grade I	1	Referent	0.001
		Grade II	8.125	1.113‐59.311	
		Grade III	13.453	1.869‐96.861	
		Grade IV	22.712	2.633‐195.932	
	T	T1	1	Referent	<0.001
		T2	1.493	1.001‐2.226	
		T3	3.024	1.844‐4.961	
		T4	2.704	1.671‐4.376	
	N	N0	1	Referent	<0.001
		N1	2.249	1.484‐3.411	
		N2	3.763	2.355‐6.012	
		N3	6.902	4.183‐11.389	
PD‐DCIS	Age at diagnosis (years)	Mean: 62.81 y	1.084	1.052‐1.116	<0.001
	T	T0	1	Referent	0.005
		T1	0.820	0.271‐2.476	
		T2	4.027	1.420‐11.417	
		T3	6.674	1.507‐29.560	
		T4	0.418	0.037‐4.730	
	N	N0	1	Referent	<0.001
		N1	6.320	1.217‐32.817	
		N2	27.077	5.035‐145.623	
		N3	28.109	3.649‐216.518	

Abbreviations**:** AJCC, American Joint Committee on Cancer; DCIS, ductal carcinoma in situ; IDC, invasive ductal carcinoma; NAC, nipple‐areola complex; PD‐DCIS, Paget disease with ductal carcinoma in situ; PD‐IDC, Paget disease with invasive ductal carcinoma

#### 3.2.3 Survival analyses for matched cohorts

**Figure 2:**
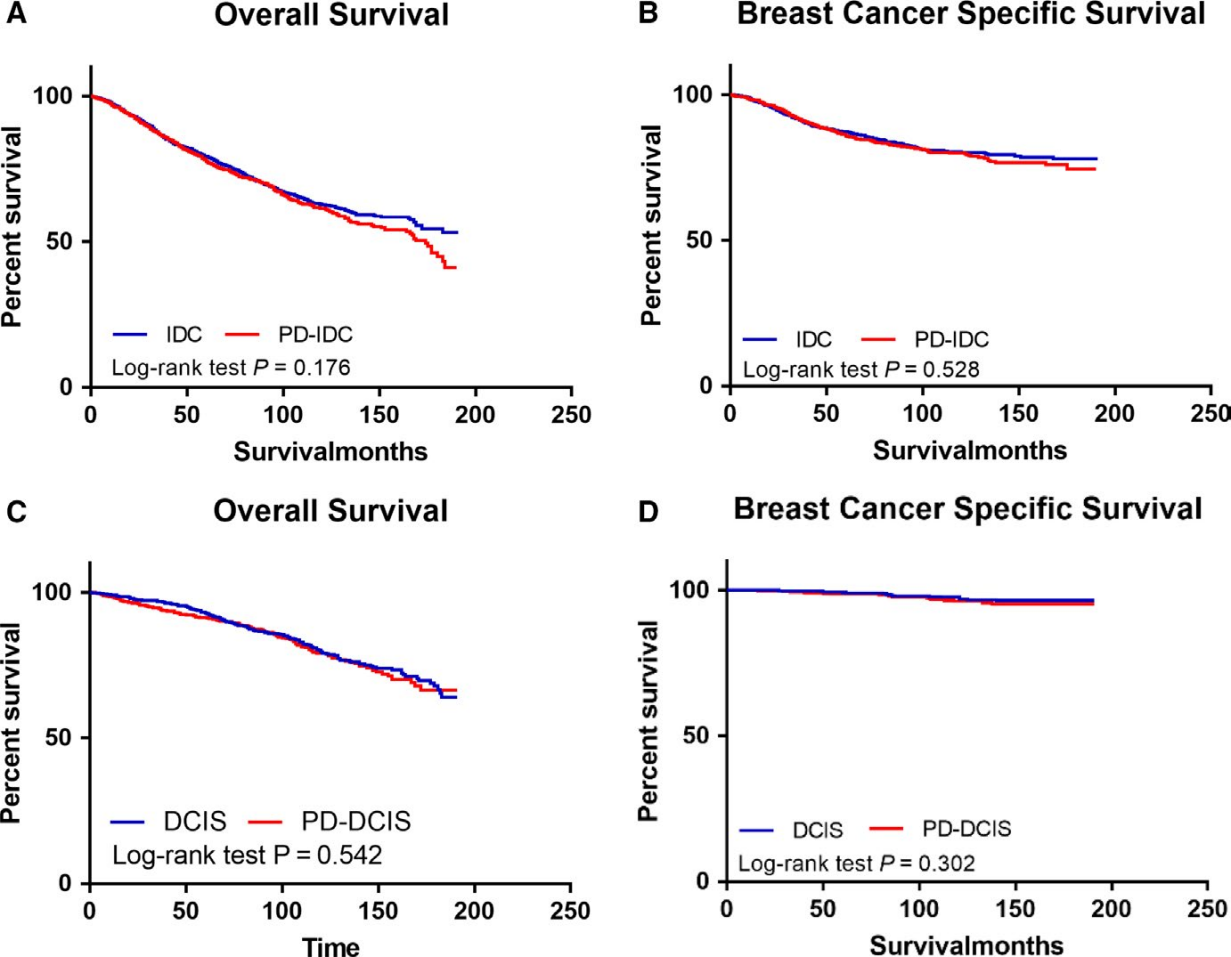
Kaplan‐Meier curves after matched for tumor characteristics and treatment. (A) overall survival of PD‐IDC group and IDC group. (B) disease‐specific survival of PD‐IDC group and IDC group. (C) overall survival of PD‐DCIS group and DCIS group. (D) disease‐specific survival of PD‐DCIS group and DCIS group

Using the PSM program, we matched two groups by adjusting for age, tumor location, tumor grade, T stage, N stage, ER status, HER2/neu status, and type of therapy (surgery, radiation, and chemotherapy)(Supplemental file‐PS matching). Then, Kaplan‐Meier analyses were conducted to study the effect of PD on survival. In cohort 1, both OS and BCSS showed no significant differences (*P* = 0.564 and *P* = 0.189, respectively) between the PD‐IDC group and the IDC group after matching tumor characteristics and treatment approaches (Figure 2A and 2). Similarly, no significant differences in OS and BCSS were found (*P* = 0.542 and *P* = 0.302, respectively) between the PD‐DCIS group and the DCIS group in cohort 2 (Figure 2C and 2).

#### 3.2.4 Comparison among the three types of DCIS

A comparison between the two types of PD‐DCIS (“Paget disease in situ and intraductal carcinoma [8543/2]” and “Paget disease and intraductal carcinoma [8543/3]”) and DCIS was conducted. The results showed that the 8543/3 group displayed a higher histological grade (66.2% of patients with 8543/3‐coded disease vs 57.1% of patients with 8543/2‐coded disease had Grade III/IV lesions, *P* = 0.036) and more advanced AJCC stage (31.8% of patients with 8543/3‐coded disease vs 0% of patients with 8543/2‐coded disease had stage I‐III cancer, *P* < 0.001) (Table S1). The survival comparison of the three types of DCIS is presented in Figure S1 and shows that the prognoses of the groups were significantly different, with the 8543/3 group having the worst prognosis. After matching tumor characteristics and treatment approaches, there were no differences in both OS and BCSS among the three groups (Figure S2).

## 4 DISCUSSION

In the present study, we analyzed the clinicopathological characteristics and survival outcomes of PD with underlying breast cancer by comparing PD with underlying breast cancer to breast cancer without concomitant PD. Our findings suggest that PD‐DCIS and PD‐IDC have poor tumor characteristics, including high‐histological grades, advanced AJCC stages, low HR‐positive ratios, and high HER2‐positive ratios. Moreover, our results confirmed that concurrent PD was associated with worse OS and BCSS than breast cancer without PD in both IDC and DCIS patients. In addition, we also found that contrary to popular belief, the HR status, HER2 status and combined molecular subtype could not affect the prognosis of breast cancer with PD. After matching tumor characteristics and treatment approaches, we demonstrated that PD appeared to alter the prognosis of DCIS but not that of IDC. Finally, by comparative analysis, we found that the disease in some patients with 8543/3‐coded PD‐DCIS exhibited many invasive behaviors, and this disease type was associated with worse survival outcomes than the other tested diseases.

Since its discovery in the 19th century, MPD has been found to correlate with underlying breast cancer. More recently, studies have shown that most MPD is accompanied by malignancy in the breast, which provides strong evidence for the generally accepted epidermotropic pathogenesis theory, which posits that ductal cancer cells migrate along the basal membrane of the nipple to form Paget cells.^12^ Nevertheless, our research suggested that not only PD‐IDC but also PD‐DCIS differed dramatically from the corresponding breast cancer without PD in terms of tumor characteristics and survival outcomes, hinting at another pathogenesis theory that posits that MPD develops in situ in the major lactiferous sinuses.^6^, ^7^, ^8^, ^9^ Further genomic and proteomic studies may be needed to illustrate more of the intrinsic properties of PD and to explore the cell source and origin site of PD.

In the SEER database, there are two types of PD‐DCIS, “Paget disease in situ and intraductal carcinoma (8543/2)” and “Paget disease and intraductal carcinoma (8543/3),” which were previously thought to be the same disease. A prior study with a limited number of subjects compared MPD with dermal invasion (invMPD) to non‐invMPD and found that dermal MPD invasion did not predict regional lymph node metastasis or a poor prognosis.^13^ The newest National Comprehensive Cancer Network (NCCN) guidelines do not distinguish the two types of PD‐DCIS either and instead classify them as noninvasive breast cancer.^14^ However, our results indicated that only the PD in situ and intraductal carcinoma (PDIS‐DCIS, 8543/2) group conformed to the characteristics of noninvasive cancer, including a negative lymph node status and good survival outcomes, while the PD and intraductal carcinoma (8543/3) group demonstrated strong invasiveness, as indicated by a positive lymph node status and poor prognosis, which is consistent with the results of another prior study.^7^ Given that PDIS‐DCIS accounts for only a small proportion of DCIS with PD patients (28.1% in this study), it is necessary to differentiate these diagnoses and consider more aggressive treatment, such as surgical axillary staging and chemotherapy, for the 8543/3 group.

In agreement with previous studies, this study verified that PD‐IDC and PD‐DCIS have a more negative HR ratio and more positive HER2 ratio than the corresponding cancers without PD.^6^, ^7^, ^8^, ^9^, ^15^, ^16^ Surprisingly, our univariate and multivariate Cox regression analyses showed that the HR status, HER2 status, and the combined molecular subtype were irrelevant to the prognoses of both PD‐IDC and PD‐DCIS. However, only the patients diagnosed after 2010 had a HER2 status and molecular subtype record in the SEER database, which might affect the accuracy of the Cox regression analysis. Nevertheless, as the HR status of most subjects was complete (89.5% of the PD‐IDC patients and 61.7% of the PD‐DCIS patients), its abnormal relationship with prognosis could not be explained by a systematic error. To the best of our knowledge, this is the first time this phenomenon has been described in a large population‐based study, and more correlative data are needed to prove this observation and illustrate the causation of the phenomenon.

More than two‐thirds of breast cancers are ER‐positive, while the rate in PD is much less than that. In SEER database only about half of MPD express ER. Several previous studies reported MPD expresses ER only in 10% of cases.^17^, ^18^ Similarly, the ER‐positive rate in extramammary Paget's disease(EMPD) was demonstrated at the range of 4%‐19%.^18^, ^19^ On the other hand, both MPD and EMPD had a relatively high rate of androgen receptor (AR) expression and antiandrogen therapy was an effective treatment for EMPD.^19^, ^20^ The different expression pattern of steroid hormone receptors suggests that maybe ER expression is not so important in MPD as in other types of breast cancer, which may be one explanation for our surprised finding. So far, there is no such study that specifically focused on the relation between molecular subtype and prognosis as well as the effect of hormone therapy and anti‐Her2 therapy in MPD. In SEER database, most of the Her2 status is unknown and the data of hormone therapy and anti‐Her2 therapy were not available, therefore, further studies are needed to confirm our unexpected discoveries and explore the causation of them.

After adjusting for other tumor characteristics and treatment approaches, we found that PD alone was not associated with inferior survival both in IDC and DCIS,^10^ supporting the consensus that the treatment strategy for MPD should be determined by the underlying breast cancer.^4^, ^14^, ^21^, ^22^ Due to the high rate of false‐negative imaging findings and high incidence of multicentric or multifocal in situ and invasive carcinomas, the traditional surgery for PD is mastectomy with or without axillary dissection.^12^ In this study, the univariate Cox regression analysis results showed that partial mastectomy was associated with a better prognosis in PD‐IDC despite no significant difference in the multivariate analysis, and there was no significant difference between partial mastectomy and mastectomy in the PD‐DCIS survival analysis. Therefore, breast‐conserving therapy is a feasible treatment option in selected patients.^23^ In addition, surgical axillary lymph node staging is a standard treatment for PD‐IDC, but its use is controversial in PD‐DCIS.^14^, ^24^ Based on our finding that some PD‐DCIS patients were lymph node positive, the consideration of surgical axillary staging is necessary. Regarding systemic adjuvant therapy, there have been few investigations and studies in PD, and the current consensus is to refer to the approach for the corresponding breast cancer without PD. In view of our surprising finding that the HR status and the HER2 status were irrelevant to the survival outcomes of PD, further study is needed to determine appropriate systemic treatment approaches.

Our study is not devoid of limitations. First, SEER database records do not contain data on HER2 status and molecular subtype until 2010, which may represent a selection bias in the survival analysis. Second, owing to the excellent prognosis of PD‐DCIS, only 36 of the 1305 PD‐DCIS patients in this study died of breast cancer, which may influence the reliability of the survival analysis. Third, the lack of information regarding systemic adjuvant treatments, particularly hormonal therapies and Her2‐targeted therapies, makes it difficult to further investigate the observation that the HR status and the HER2 status were not associated with the survival outcomes of PD.

In conclusion, we found that PD‐IDC and PD‐DCIS had more aggressive tumor characteristics and worse survival than the corresponding breast cancer without PD. Nevertheless, after adjusting for tumor characteristics and treatment approaches, PD was related to poor prognosis in only DCIS. Moreover, some PD‐DCIS cases exhibited many invasive behaviors and were associated with higher mortality. Finally, contrary to popular belief, the HR status and the HER2 status could not affect the prognosis of breast cancer with PD.

## 5 DATA SHARING AND DATA ACCESSIBILITY

National Cancer Institute. Surveillance, Epidemiology, and End Results (SEER) Program (www.seer.cancer.gov) SEER*Stat Database: Incidence ‐ SEER 18 Regs Custom Data (with additional treatment fields), Nov 2017 Sub (1973‐2015 varying) ‐ Linked To County Attributes ‐ Total US, 1969‐2016 Counties, National Cancer Institute, DCCPS, Surveillance Research Program, released April 2018, based on the November 2017 submission. Available at: https://seer.cancer.gov/data/, Accessed November 13, 2018.

## CONFLICT OF INTEREST

All authors declare that they have no conflict of interest.

## AUTHOR CONTRIBUTIONS

Shijing Chen was involved in final data analysis and manuscript writing. Huaquan Chen was involved in data collection and management. Ying Yi, Xuemei Jiang, Hai Lei, Xue Luo, Yu Chen, Sha Liu, and Dan Yuan were assisted in the data collection and analysis. Xinjian Jia was involved in statistical analysis and quality control. Junyan Li was involved in study design and responsible for the entire research project.
