# Supplementary figures and images for: Comparative study of breast cancer with or without concomitant Paget disease: An analysis of the SEER database
Source: Cancer Med. 2019 May 28;8(8):4043–54. doi: 10.1002/cam4.2242 (PMC6639179; doi:10.1002/cam4.2242)

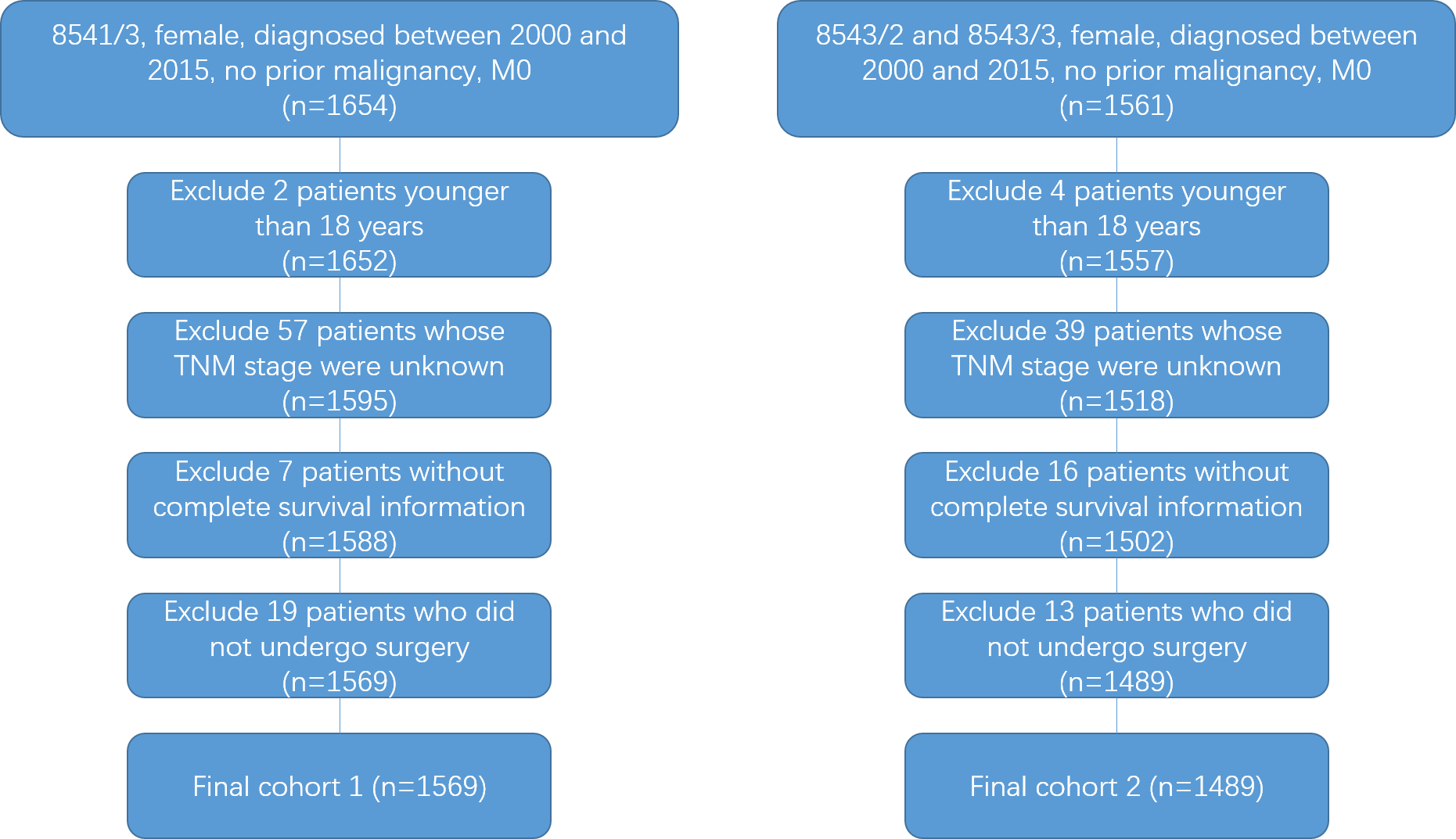

Supplement: Supplementary file 1 [file CAM4-8-4043-s001.tif]

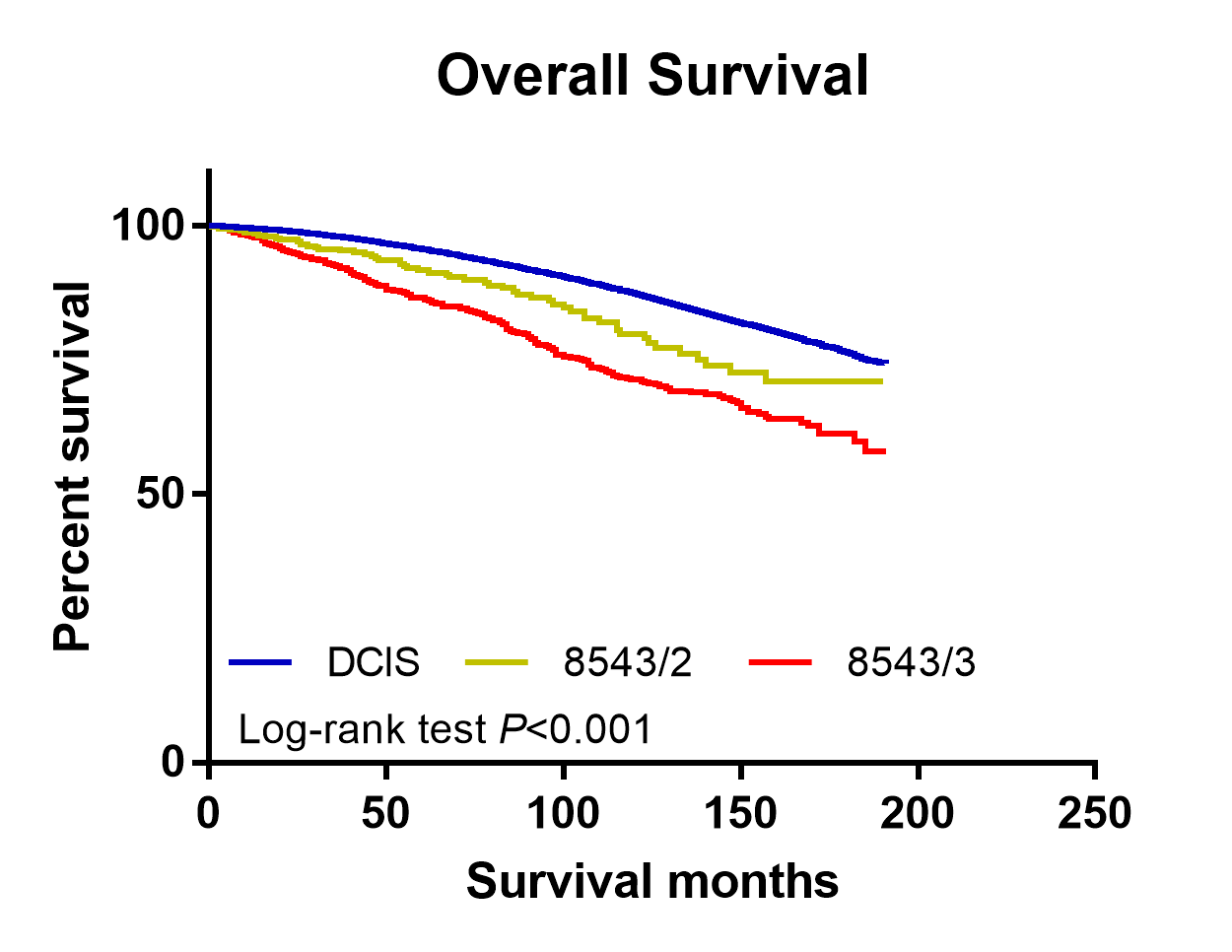

Supplement: Supplementary file 2 [file CAM4-8-4043-s002.tif]

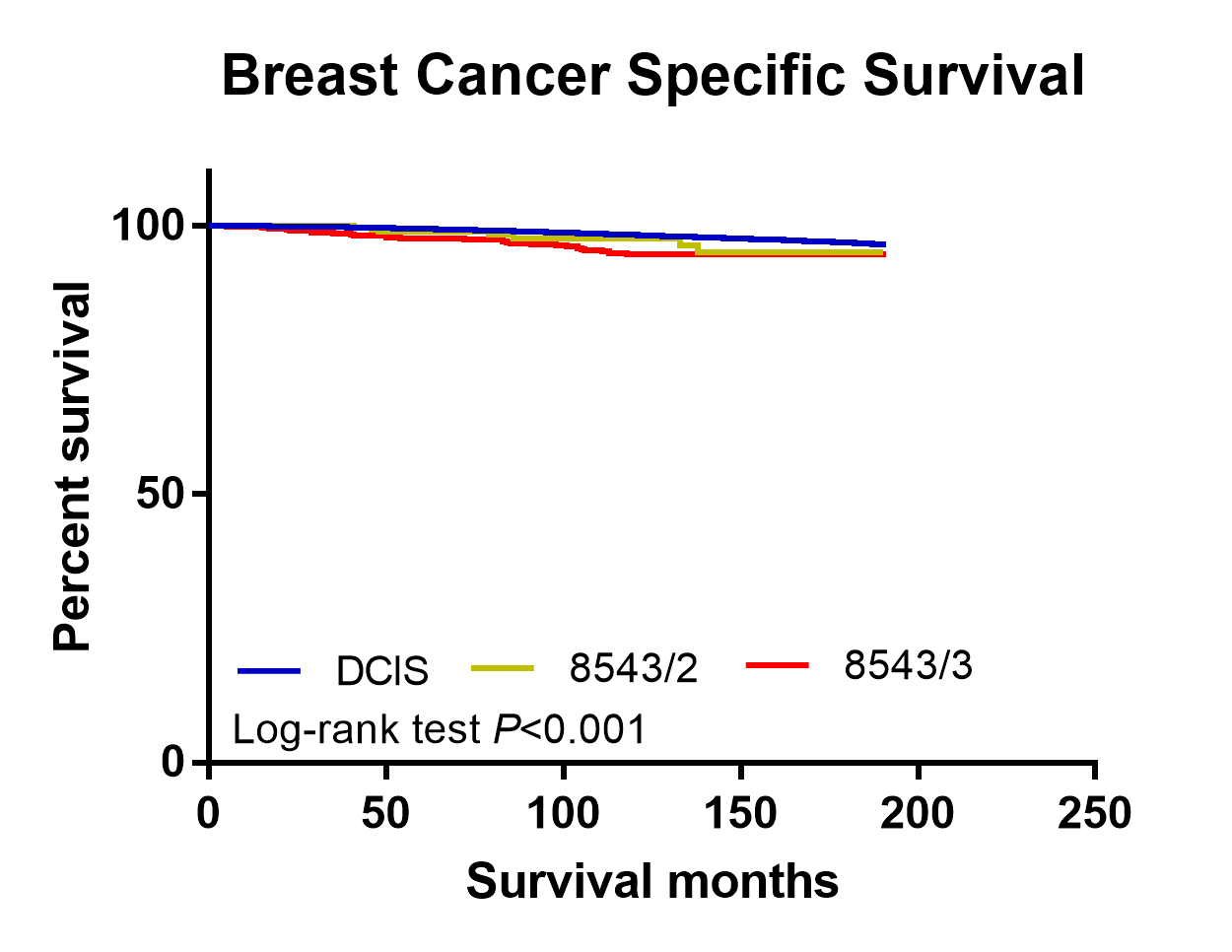

Supplement: Supplementary file 3 [file CAM4-8-4043-s003.tif]

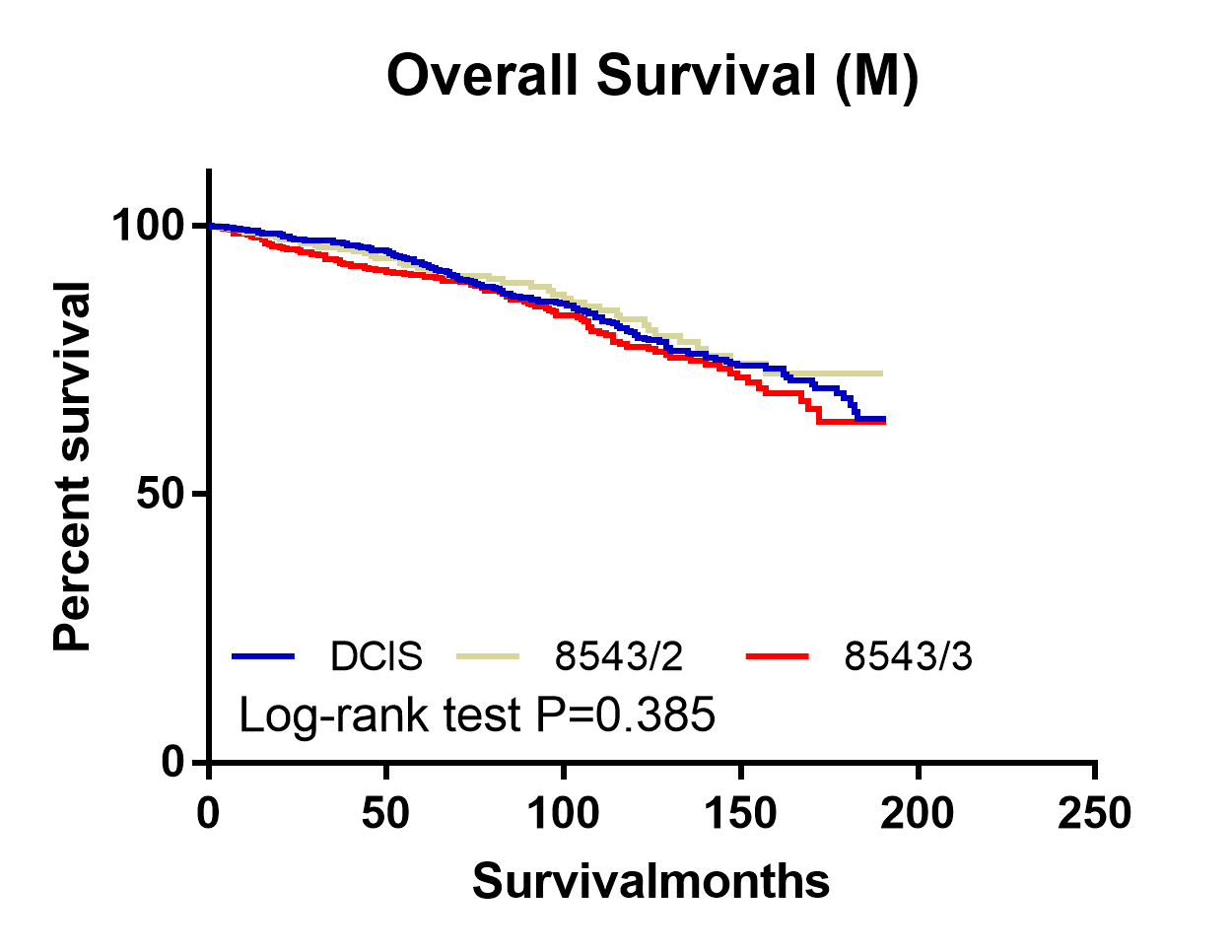

Supplement: Supplementary file 4 [file CAM4-8-4043-s004.tif]

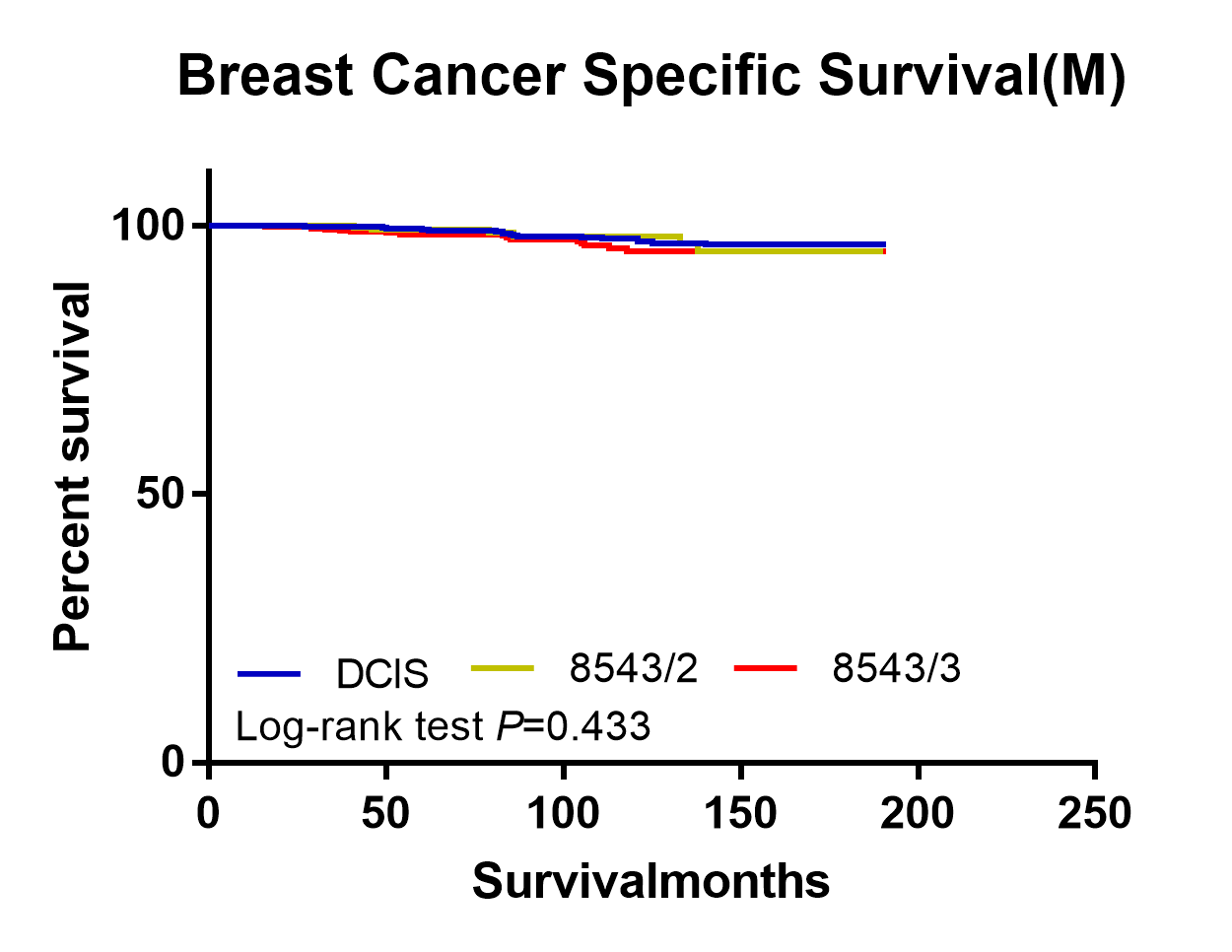

Supplement: Supplementary file 5 [file CAM4-8-4043-s005.tif]
